# Comprehensive Assessment of Milk Composition in Transgenic Cloned Cattle

**DOI:** 10.1371/journal.pone.0049697

**Published:** 2012-11-21

**Authors:** Ran Zhang, Chengdong Guo, Shunchao Sui, Tian Yu, Jianwu Wang, Ning Li

**Affiliations:** 1 State Key Laboratory of Agrobiotechnology, China Agricultural University, Beijing, China; 2 Wuxi Kingenew Biotechnology Co., Ltd., Wuxi, Jiangsu Province, China; 3 Beijing GenProtein Biotech Company Ltd., Beijing, China; University of Connecticut, United States of America

## Abstract

The development of transgenic cloned animals offers new opportunities for agriculture, biomedicine and environmental science. Expressing recombinant proteins in dairy animals to alter their milk composition is considered beneficial for human health. However, relatively little is known about the expression profile of the proteins in milk derived from transgenic cloned animals. In this study, we compared the proteome and nutrient composition of the colostrum and mature milk from three lines of transgenic cloned (TC) cattle that specifically express human α-lactalbumin (TC-LA), lactoferrin (TC-LF) or lysozyme (TC-LZ) in the mammary gland with those from cloned non-transgenic (C) and conventionally bred normal animals (N). Protein expression profile identification was performed, 37 proteins were specifically expressed in the TC animals and 70 protein spots that were classified as 22 proteins with significantly altered expression levels in the TC and C groups compared to N group. Assessment of the relationship of the transgene effect and normal variability in the milk protein profiles in each group indicated that the variation in the endogenous protein profiles of the three TC groups was within the limit of natural variability. More than 50 parameters for the colostrum and mature milk were compared between each TC group and the N controls. The data revealed essentially similar profiles for all groups. This comprehensive study demonstrated that in TC cattle the mean values for the measured milk parameters were all within the normal range, suggesting that the expression of a transgene does not affect the composition of milk.

## Introduction

The rapid development of transgenic technology has led to the generation of a broad spectrum of transgenic cloned animals for agricultural and biomedical use [1], [2]. Among numerous applications, expressing recombinant human proteins with benefits for human health and nutrition has become a reality (http://www.gtc-bio.com/; http://www.pharming.com/). However, some aspects of the process of cloning transgenic animals, such as epigenetic reprogramming, exogenous gene insertion and pleiotropy, have increased the attention paid to the risks of consuming these foods and to the health of the animals [3]–[5]. Therefore, studying the composition of the products of these transgenic cloned livestock is important to demonstrate whether there is a risk associated with foods produced from these animals compared with food from conventionally bred animals.

Since 2001, the FDA has conducted an intensive evaluation that includes the examination of the safety of food from cloned animals and the risks of cloning to animal health. The accumulated data indicate that the gross composition of milk and meat from cloned animals is within the normal range and is as safe as milk and meat from conventionally bred cattle [6]–[8]. Meanwhile, similar considerations have been given to the safety of food produced from transgenic animals. Transgenesis and cloning belong to two different risk groups because new transgenes are introduced into the genome of transgenic animals but not in cloned animals. Therefore, the analysis of the risk of consuming foods from transgenic animals should focus on characterization of the expected products of the transgene and any other unintended changes [9]. To date, very limited published information is available on the composition of food products derived from transgenic cloned animals. One study that examined the production of milk containing higher than normal levels of bovine β-casein and κ-casein revealed that the nutritional composition of the milk from the transgenic cloned cattle and conventionally bred cattle was similar [10].

However, some unanswered concerns remain. Whether the protein profiles of milk from transgenic cloned cattle are altered by the cloning technology or by the expression of exogenous human genes in bovine mammary epithelial cells, and the mechanism by which such changes might occur, is unknown. Technological advances in proteomics have allowed an increased understanding and characterization of milk proteins. Previous proteomic studies have focused on the identification of milk whey proteins and have the advantage of being able to detect, identify and characterize a large number of milk proteins simultaneously, including low-abundance proteins [11]–[13]. Thus, current proteomic technology is an essential component of addressing the food safety concerns associated with transgenic technology and of making the milk from transgenic cloned animals more attractive to consumers.

Previously, we described the generation of three lines of transgenic cloned cattle that specifically express human α-lactalbumin (TC-LA), lactoferrin (TC-LF) or lysozyme (TC-LZ) in milk as a result of the integration of a specific transgene into the genome and were cloned by subsequent SCNT [14]–[16]. The major changes observed between milk from these animals and milk from conventionally bred animals were the high levels of human milk proteins. Thus, the milk of these transgenic animals has very different properties from that of conventionally bred animals and provides a unique model for evaluating the effects of exogenous transgenes on the profile of the endogenous milk proteins. The objective of this study was to examine differences in the milk proteome and other important milk components in the colostrum and mature milk from three transgenic cloned cattle lines. This comprehensive study provides unequivocal evidence that the expression of a transgene does not affect the composition of milk, and these results may assist with the assessment of the safety of food derived from transgenic cloned animals.

## Materials and Methods

### TC Cattle and Matched Breed Controls

The three different lines of transgenic cattle expressing approximately 0.01 mg/mL hLZ (n = 10), 1.5 mg/mL hα-LA (n = 4), or 3.5 mg/mL hLF (n = 3), as described previously [14]–[16], were used for the milk composition analysis, with the C (n = 3) and N animals (n = 9) used as controls. In briefly, the concentration of the recombinant proteins was assayed by ELISA using Human Lactoferrin ELISA Kit (Bethyl, Montgomery, TX, USA), Human LYZ ELISA Kit (Abnova, Taiwan) and Human LALBA ELISA Kit (Abnova, Taiwan), respectively, according to the manufacturer’s instructions. The absorbance of the product was measured at 450 nm using a model 550 microplate reader (Bio-Rad, Hercules, CA). All TC cattle lines and C cattle were produced by SCNT, with the C control cattle being from the unmodified donor nuclear cell lines that were used to produce the TC cattle following the introduction of the transgenes. In addition, the Holstein cow breed N cattle had a genetic background similar to that of the transgenic cloned cattle. The cattle were similar in age and lactation period and were housed under the same conditions.

### Milk Sample Collection

The protocol was approved by the Institutional Animal Care and Use Committee of China Agricultural University (ID: SKLAB-2010-05-01). The colostrum was obtained during the initial three days of lactation and mature milk was obtained on the 30^th^, 60^th^ and 90^th^ day after lactation. Milk was collected from the cows twice daily and was pooled to form one daily sample. A portion of the samples was used for the analysis of the milk nutrient composition. The remaining portion was centrifuged at 2500×*g* for 30 min at 4°C to obtain the skim milk fraction and subsequently ultracentrifuged at 150,000×*g* for 1 h at 4°C to remove the casein micelles. The supernatant (whey fraction) of each cow per day was collected and stored at −80°C for ELISA analysis. For the proteomics analysis, three days of mature whey samples from each cow was pooled together to generate one individual sample and then individual samples in the same groups (TC-LZ, TC-LA, TC-LF, C and N) were pooled again to generate one group sample according to the equal protein mass (three days of whey samples from nine control cows were pooled and then analyzed separately for HCA and PCA analysis).

### 2D SDS-PAGE

The whey protein was precipitated using a 2D Clean-UP kit (GE Healthcare). Whey proteins (1 mg) were loaded onto IPG strips (linear, 24 cm length, pH 4–7) by overnight rehydration at 20°C. The IEF was performed using IPGphor III (GE Healthcare) by gradually increasing the voltage. SDS-PAGE was performed using an Ettan DALT System (GE Healthcare) and the protein spots were visualized by “blue-silver” staining. The image analysis was performed using the Image Master 2D Platinum 6.0 software (GE Healthcare). Student’s *t*-tests were performed on the proteins showing greater than 2-fold changes, and *p*≤0.05 were considered to be significant.

### LC-MS/MS Analysis

The in-solution tryptic digestion proteins (200 µg) were analyzed using 2D-Nano-LC-ESI-MS/MS, performed on a nano Acquity UPLC (Waters) connected to a LTQ Orbitrap XL mass spectrometer (Thermo Fisher). Each scan cycle consisted of one full MS scan in profile mode followed by five MS/MS scans in centroid mode. The acquired MS/MS spectra were used to search against a non-redundant protein Bovidae database using the SEQUEST program in the BioWorks™ 3.3.1 software suite with the following parameters: the mass tolerance of 1.4 Da for precursor ions and 1.0 Da for fragment ions, allowing two missed cleavage. The false discovery rate was less than 1%, which was calculated using a database containing reversed sequences. The peptide identifications were filtered by PeptideProphet with a confidence level of 95% and protein identifications were accepted with greater than 99% probability [17].

### MALDI-TOF MS/MS Analysis

In-gel digestion was performed using standard protocols [18]. The peptides were analyzed on a 4700 MALDI-TOF/TOF mass spectrometer (Applied Biosystems) operated in reflector positive mode, followed by MS/MS of eight most-intense peptide ions. The acquired MS/MS spectra were used to search against a BOVINEnr database in the MASCOT software with the following parameters: a peptide tolerance of 100 ppm, a fragment tolerance of 0.6 Da, allowing one trypsin missed cleavage.

### Quantitative Analysis of Major Whey Proteins

The amount of seven major bovine whey proteins (β-LG, α-LA, BSA, IgG, IgA, IgM and transferrin) in the colostrum and mature milk from the five groups of animals was determined using an enzyme-linked immunosorbent assay (ELISA) kit (Bethyl, Montgomery, TX, USA) according to the manufacturer’s instructions. The absorbance of the product was measured at 450 nm using a model 550 microplate reader (Bio-Rad, Hercules, CA).

### Nutrient Composition Analysis

The whole-milk samples were delivered to the Beijing Research Institute for Nutritional Resources, which establishes the criteria and methods for the analysis of large quantities of biochemical and nutritional components. More than 50 nutritional components were analyzed and they include 18 kinds of amino acids, 4 kinds of total fatty acids as well as 16 kinds of specific fatty acid profiles, mineral (K, Na, Ca, Mg, Fe, Zn, Se and P) and vitamin contents (vitamin A, vitamin B1, vitamin B2, Vitamin B3, vitamin B5, vitamin B12 and vitamin C). The entire composition was measured according to the national food safety standard raw milk issued by China’s Ministry of Health (GB19301-2010). All values show standard deviation and statistically analyzed using *t*-test (*p<0.05).

## Results

### Overview of Milk Whey Proteome and Identification of Specifically Expressed Proteins in the TC Groups

**Figure 1:**
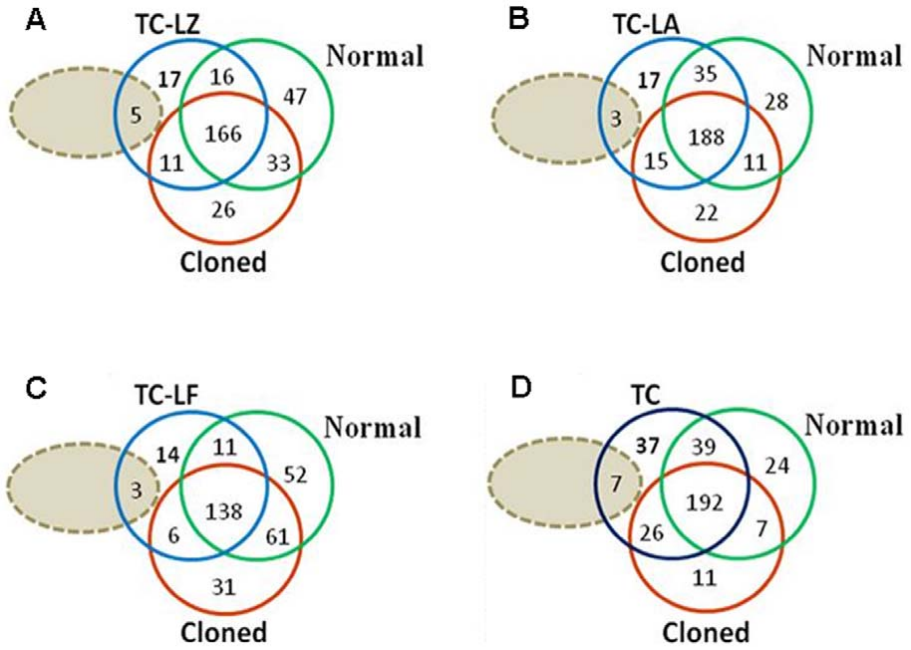
Venn diagrams representation of whey protein profiles comparison of the TC-LZ (A), TC-LA (B), TC-LF (C) and all TC (D) groups were created with respect to the C and N groups, respectively. The gray ellipse indicates the proteins reported to be present in bovine milk in previously studies. The blue, green and red circles indicate the protein sets of the TC, C and N groups, respectively.

**Figure 2:**
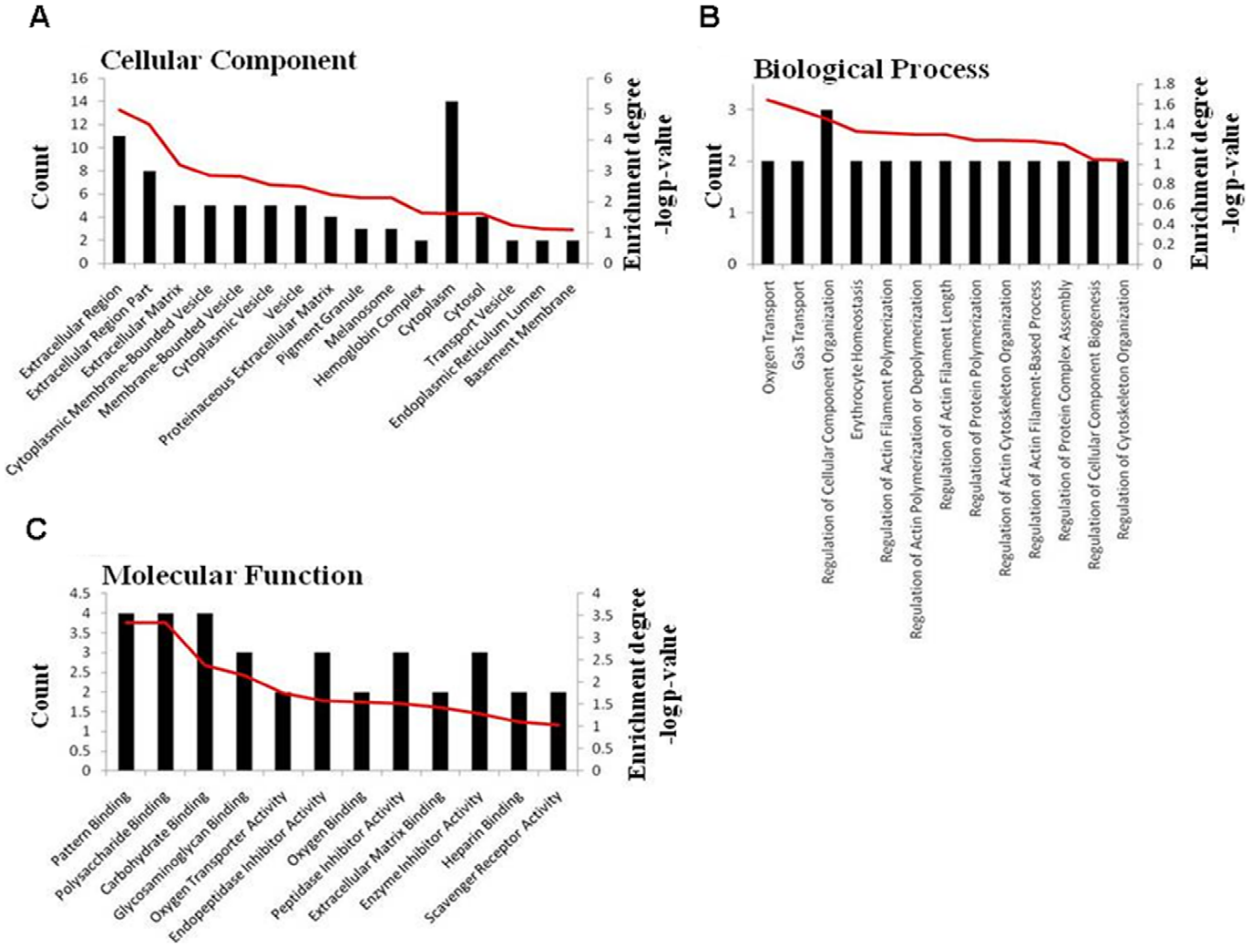
Functional distribution and enrichment of specifically expressed proteins classified according to cellular component (A), biological process (B) and molecular function (C) in the TC groups, respectively. The red curves indicate the degree of functional enrichment of a particular gene.

The milk protein compositions of each group were compared comprehensively using 2D-Nano-LC-MS/MS. Database searches using the fragmentation spectra as experimental material identified unique proteins from distinct peptides, and 215, 258, 172, 236 and 262 proteins were identified in the TC-LZ, TC-LA, TC-LF, C and N groups, respectively. Venn diagrams of the protein profiles of the TC-LZ, TC-LA, TC-LF and C groups were created with respect to the N group. The TC-LZ had 182 proteins in common with the N group, and 33 and 80 proteins were unique to the TC-LZ and N groups, respectively (Figure 1A). A similar distribution was found for the TC-LA, TC-LF and C groups (Figure 1B–1D). Altogether, 44 proteins were identified that were exclusive to the TC groups, 7 of which had been previously confirmed to be present in milk from cattle of the background strain using similar technological strategies [19]–[21]. The TC and C groups had 26 proteins in common that were not detected in the N group (Figure 1D). To further examine the specifically expressed proteins, the 37 newly identified proteins were categorized according to their cellular component, biological process or molecular function as annotated in the Gene Ontology (GO) database (Table 1 and Figure 2). Our results indicate that the majority of the proteins detected were either completely or partially extracellularly localized, as expected for milk proteins. When organized according to biological activity, most proteins were found to be involved in oxygen transport. Functionally, the majority of proteins identified were involved in pattern binding, polysaccharide or carbohydrate binding.

**Table 1:** List of the proteins specifically expressed in the transgenic groups.

No.	Protein Name	Accession No.	LZ	LA	LF	MW (kDa)	pI	Cellular Component	Biological Process	Molecular Function
1	MHC CLASS I HEAVY CHAIN (FRAGMENT)	IPI00954418.1	N*	N	D^†^	19.3	4.79	membrane; MHC class I protein complex	immune response; antigen processing and resentation	U^‡^
2	SERPINA3-2	IPI00930024.1	N	N	D	46.2	5.66	extracellular space; cytoplasmic vesicle	U	serine-type endopeptidase inhibitor activity
3	22 kDa PROTEIN	IPI00907745.1	N	D	N	22.4	9.71	U	U	U
4	SIMILAR TO CYCLOPHILIN B (FRAGMENT)	IPI00883489.1	N	D	N	11.0	9.74	U	protein folding	peptidyl-prolyl cis-trans isomerase activity
5	C8G PROTEIN	IPI00876781.1	N	D	N	25.9	11.1	U	transport	binding; transporter activity
6	ERAP1 PROTEIN	IPI00867238.1	N	N	D	10.7	5.91	cytoplasm	proteolysis; positive regulation of angiogenesis	metallopeptidase activity; aminopeptidase activity; zinc ion binding
7	GPR56 PROTEIN	IPI00847084.1	D	N	D	77.0	8.80	integral to membrane; plasma membrane	cell adhesion; brain development neuropeptide signaling pathway	protein binding; G-protein coupled receptor activity
8	KRT6A PROTEIN	IPI00845184.1	D	N	N	60.8	8.28	keratin filament	U	structural molecule activity
9	51 kDa PROTEIN	IPI00841672.1	D	N	N	51.2	4.90	U	U	U
10	13 kDa PROTEIN	IPI00841591.2	N	D	N	13.2	8.66	U	U	U
11	39 kDa PROTEIN	IPI00841003.2	N	D	N	38.8	8.73	extracellular region	U	U
12	31 kDa PROTEIN	IPI00840962.2	D	N	D	30.7	4.44	extracellular region	U	U
13	SIMILAR TO CALSYNTENIN-1, PARTIAL	IPI00838241.2	N	D	N	107	4.73	U	U	U
14	174 kDa PROTEIN	IPI00838035.2	N	N	D	174	6.52	U	U	U
15	SIMILAR TO KERATIN 2	IPI00824847.1	D	N	N	64.3	8.56	U	U	U
16	SIMILAR TO CD5 MOLECULE LIKE	IPI00824588.2	N	N	D	82.7	5.19	U	U	U
17	MHC CLASS I ANTIGEN (FRAGMENT)	IPI00788543.1	N	N	D	19.9	5.03	membrane; MHC class I protein complex	antigen processing and presentation; immune response	U
18	NAGLU PROTEIN	IPI00717554.3	D	N	N	74.1	5.81	U	U	U
19	HEMOGLOBIN SUBUNIT BETA	IPI00716455.1	N	D	N	16.0	7.01	hemoglobin complex	transport; oxygen transport	metal ion binding; oxygen binding; heme binding
20	CYSTEINE-RICH SECRETORY PROTEIN LCCL DOMAIN -CONTAINING 2	IPI00715724.4	N	D	N	55.6	8.67	extracellular matrix; transport vesicle; extracellular region	extracellular matrix organization	heparin binding
21	ACTIN-RELATED PROTEIN 2/3 COMPLEX SUBUNIT 2	IPI00715364.1	D	N	N	34.3	6.84	cytoskeleton; cell projection cytoplasm	regulation of actin filament polymerization	actin binding
22	HEMOGLOBIN SUBUNIT ALPHA	IPI00710783.2	N	D	N	15.2	8.06	hemoglobin complex	oxygen transport	heme binding; oxygen binding; oxygen transporter activity
23	METALLOPROTEINASE INHIBITOR 1	IPI00709084.1	N	D	N	23.0	8.46	basement membrane	erythrocyte maturation; negative regulation of catalytic activity	metal ion binding; metalloendopeptidase inhibitor activity
24	ENDOPIN 2C	IPI00705594.1	D	N	D	46.7	5.98	U	U	serine-type endopeptidase inhibitor activity
25	TESTICAN 1	IPI00705387.2	D	D	N	49.4	5.34	proteinaceous extracellular matrix	signal transduction	calcium ion binding
26	RPE-SPONDIN	IPI00704612.4	N	D	N	29.3	7.71	extracellular region	immune response	scavenger receptor activity; polysaccharide binding
27	RIBONUCLEASE T2	IPI00704364.3	N	D	N	43.5	9.98	U	U	RNA binding; ribonuclease T2 activity
28	VON WILLEBRAND FACTOR A DOMAIN -CONTAINING PROTEIN 1	IPI00701880.2	D	N	N	43.7	8.71	basement membrane; interstitial matrix; ribosome	extracellular matrix organization; translation	structural constituent of ribosome
29	BIGLYCAN	IPI00697081.2	N	D	N	41.6	6.83	proteinaceous extracellular matrix; sarcolemma; transport vesicle	peptide cross-linking via chondroitin 4-sulfate glycosaminoglycan	glycosaminoglycan binding; extracellular matrix binding
30	OSTEOPONTIN-K	IPI00696774.1	D	N	D	31.0	4.56	extracellular region	cell adhesion; ossification	protein binding
31	111 kDa PROTEIN	IPI00695031.5	D	N	D	111.7	5.26	U	U	ATP binding
32	83 kDa PROTEIN	IPI00694678.3	D	N	D	83.2	6.15	extracellular region	U	endopeptidase inhibitor activity
33	GELSOLIN	IPI00694255.2	D	N	D	80.7	5.54	cytoplasm; extracellular region cytoskeleton	actin filament capping; cilium morphogenesis	actin binding; metal ion binding
34	ENDOPLASMIN	IPI00692865.2	D	D	N	92.7	4.77	cytosol; endoplasmic reticulum; melanosome	ER-associated protein catabolic process; protein folding; anti-apoptosis	RNA binding; ATP binding; virion binding
35	MFGE8 PROTEIN	IPI00689638.1	D	N	D	47.9	6.80	extracellular space	cell adhesion	phosphatidylethanolamine binding
36	DIPEPTIDYL PEPTIDASE III	IPI00686733.2	N	D	N	82.1	5.09	cytoplasm	proteolysis	dipeptidyl-peptidase activity
37	PEROXIREDOXIN-1	IPI00686092.1	D	D	N	22.2	8.59	melanosome	regulation of NF-kappaB import into nucleus	thioredoxin peroxidase activity

* Proteins not detected by LC-MS/MS in the transgenic groups (LZ, LA and LF); ^†^Proteins detected by LC-MS/MS; ^‡^No function annotation.

### Characterization of Differentially Expressed Proteins in the TC Groups

**Figure 3:**
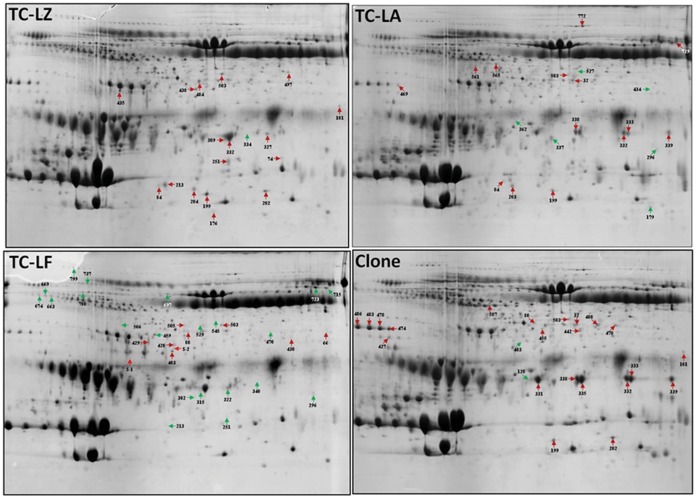
Differentially expressed protein spots with great than 2-fold changes (*p*≤0.05) in the TC and the C group compared to N group. The red and green arrows indicate the protein spots that were significantly up- and downregulated, respectively, by at least 2-fold.

**Figure 4:**
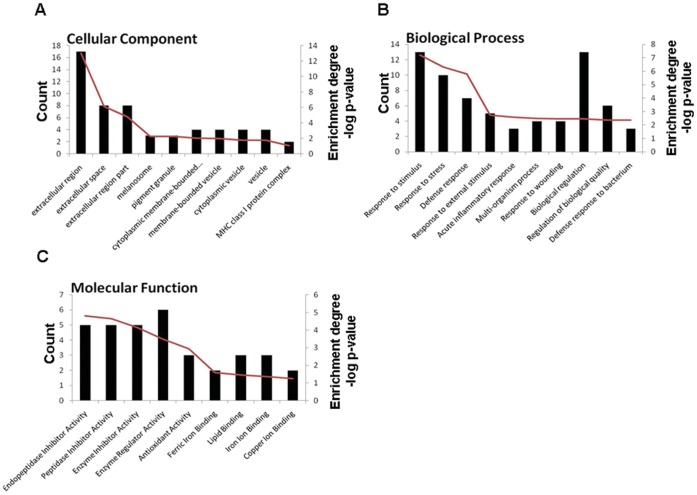
Functional distribution and enrichment of differentially expressed proteins classified according to cellular component (A), biological process (B) and molecular function (C) in the TC and the C group, respectively. The red curves indicate the degree of gene functional enrichment.

To assess whether the variation in milk protein expression profiles was due to the effect of the transgene or to natural variability between each group, the TC, C and N groups were investigated using a quantitative 2DE combined with MALDI TOF/TOF. A total of 708 protein spots were detected on 2DE, and 602 protein spots that originated from unique genes were positively identified by MALDI-TOF/TOF. Many of the spots that were not identified were for proteins of extremely low abundance. ImageMaster and statistical analyses revealed that 58 spots were significantly different between the TC and N groups and 12 spots between the C and N groups, with at least 2-fold differences (*p*≤0.05) (Figure 3). Of these 70 spots, 46 spots, which were classified into 22 types of proteins, were identified successfully. Compared with the N group, leukocyte elastase inhibitor (SERPINB1) was changed in all four groups; 10 proteins were differentially expressed in only one group whereas 7 proteins in any two groups. Lactotransferrin (LTF) was absent in the C group and upregulated in both the TC-LA and TC-LZ groups but downregulated in the TC-LF group. Cathelicidin-1 (CATHL1) was absent in the TC-LF group but upregulated in the other three groups. A complete list of the identified differentially expressed proteins is provided in Table 2, where these proteins are grouped according to GO classifications (Figure 4). In terms of biological processes, the majority of proteins belonged to the defense response; when grouped by molecular function, the proteins most commonly had endopeptidase inhibitor activity, and the majority of proteins were components of the extracellular region.

**Table 2:** List of the proteins differentially expressed in the transgenic and cloned groups compare to normal group.

No.	Group ID	Protein name	Accession No.	LZ	LA	LF	C	MW (kDa)	pI	Cellular Component	Biological Process	Molecular Function
1	32	U*	U		↑^†^		↑	U	U	U	U	U
2	66	U	U			↑		U	U	U	U	U
3	74	LACTOTRANSFERRIN	IPI00710664	↑				78.1	8.68	extracellular region; secretory granule	cellular iron ion homeostasis; defense response to bacteria	heparin binding; ferric iron binding; serine-type peptidase activity
4	80	26 kDa PROTEIN	IPI00906471			↑	↑	26.0	8.41	extracellular region	N^‡^	N
5	84	U	U	↑	↑			U	U	U	U	U
6	101	U	U	↑			↑	U	U	U	U	U
7	120	CSN2 18 kDa PROTEIN	IPI00712994				↓^§^	18.3	5.13	extracellular region	transport	transporter activity; protein binding
8	176	U	U	↑				U	U	U	U	U
9	179	26 kDa PROTEIN	IPI00906471		↓			26.0	8.41	extracellular region	N	N
10	199	CATHELICIDIN-1	IPI00717085	↑	↑		↑	17.6	8.26	extracellular region	defense response	N
11	202	CATHELICIDIN-1	IPI00717085	↑			↑	17.6	8.26	extracellular region	defense response	N
12	203	LACTOTRANSFERRIN	IPI00710664		↑			78.1	8.68	extracellular region; secretory granule	cellular iron ion homeostasis; defense response to bacteria	heparin binding; ferric iron binding; serine-type peptidase activity
13	204	U	U	↑				U	U	U	U	U
14	213	LACTOTRANSFERRIN	IPI00710664	↑		↓		78.1	8.68	extracellular region; secretory granule	cellular iron ion homeostasis; defense response to bacteria	heparin binding; ferric iron binding; serine-type peptidase activity
15	251	26 kDa PROTEIN	IPI00906471	↑		↓		26.0	8.41	extracellular region	N	N
16	296	CSN2 18 kDa PROTEIN	IPI00712994		↓	↓		18.3	5.13	extracellular region	transport	transporter activity; protein binding
17	302	U	U			↓		U	U	U	U	U
18	309	U	U	↑				U	U	U	U	U
19	315	CSN2 18 kDa PROTEIN	IPI00712994			↓		18.3	5.13	extracellular region	transport	transporter activity; protein binding
20	322	U	U	↑		↓		U	U	U	U	U
21	327	CSN2 18 kDa PROTEIN	IPI00712994	↓				18.3	5.13	extracellular region	transport	transporter activity; protein binding
22	331	U	U				↑	U	U	U	U	U
23	332	FGG 50 kDa PROTEIN	IPI00843209		↑		↑	50.2	5.45	extracellular region	signal transduction	protein binding
24	333	PIGMENT EPITHELIUM-DERIVED FACTOR	IPI00716121		↑		↑	46.2	6.56	extracellular space; extracellular matrix; melanosome	negative regulation of angiogenesis; regulation of proteolysis	serine-type endopeptidase inhibitor activity
25	334	26 kDa PROTEIN	IPI00906471	↓				26.0	8.41	extracellular region	N	N
26	335	U	U				↑	U	U	U	U	U
27	337	CSN2 18 kDa PROTEIN	IPI00712994		↓			18.3	5.13	extracellular region	transport	transporter activity; protein binding
28	338	U	U		↑		↑	U	U	U	U	U
29	339	PROSTAGLANDIN-H2 D-ISOMERASE	IPI00709683		↑		↑	21.2	6.42	Golgi apparatus; extracellular space; nuclear membrane	prostaglandin biosynthetic process; regulation of circadian sleep/wake cycle, sleep	prostaglandin-D synthase activity; fatty acid binding
30	340	CSN2 18 kDa PROTEIN	IPI00712994			↓		18.3	5.13	extracellular region	transport	transporter activity; protein binding
31	362	LACTOTRANSFERRIN	IPI00710664		↓			78.1	8.68	extracellular region; secretory granule	cellular iron ion homeostasis; defense response to bacteria	heparin binding; ferric iron binding; serine-type peptidase activity
32	403	U	U			↑	↓	U	U	U	U	U
33	420	COMPLEMENT C3	IPI00713505			↑		U	6.41	extracellular space; extracellular region	inflammatory response; complement activation alternative pathway	endopeptidase inhibitor activity
34	427	CATIONIC TRYPSIN	IPI00706427				↑	26.0	8.40	extracellular space	proteolysis; digestion	serine-type endopeptidase activity; metal ion binding
35	429	COMPLEMENT C3	IPI00713505			↑		187.3	6.41	extracellular space; extracellular region	inflammatory response; complement activation, alternative pathway	endopeptidase inhibitor activity
36	430	U	U			↑		U	U	U	U	U
37	434	U	U		↓			U	U	U	U	U
38	435	ZINC-ALPHA-2-GLYCOPROTEIN	IPI00698993	↑				33.9	5.13	extracellular region; membrane; MHC class I protein complex	immune response; antigen processing and presentation	N
39	438	BOLA-NC1	IPI00710100	↑				37.5	6.27	membrane	immune response	N
40	442	U	U				↑	U	U	U	U	U
41	455	CATIONIC TRYPSIN	IPI00706427				↑	26.0	8.40	extracellular space	proteolysis; digestion	serine-type endopeptidase activity; metal ion binding
42	459	APOLIPOPROTEIN A-IV	IPI00695965			↓		43.0	5.29	chylomicron; high density lipoprotein particle	lipid transport; lipoprotein metabolic process	lipid binding
43	469	ALPHA-1-ACID GLYCOPROTEIN	IPI00691212		↑			23.2	5.61	extracellular space	regulation of immune system process	N
44	470	U	U			↓	↑	U	U	U	U	U
45	474	ORM1 23 kDa PROTEIN	IPI00903510				↑	23.2	5.99	extracellular space	regulation of immune system process	N
46	478	ALPHA-1-ACID GLYCOPROTEIN	IPI00691212				↑	23.2	5.61	extracellular space	regulation of immune system process	N
47	480	U	U				↑	U	U	U	U	U
48	483	ALPHA-1-ACID GLYCOPROTEIN	IPI00691212				↑	23.2	5.61	extracellular space	regulation of immune system process	N
49	484	CATHEPSIN B	IPI00692061	↑				36.7	5.68	lysosome; mitochondrion	proteolysis; regulation of catalytic activity	cysteine-type endopeptidase activity
50	497	U	U	↑				U	U	U	U	U
51	503	SERPINB1 LEUKOCYTE ELASTASE INHIBITOR	IPI00710789	↑	↑	↓	↑	42.2	5.70	cytoplasm	regulation of proteolysis	serine-type endopeptidase inhibitor activity
52	505	U	U			↓		U	U	U	U	U
53	506	U	U			↓		U	U	U	U	U
54	527	ALB PROTEIN	IPI00708398		↓			69.5	5.82	extracellular space	transport	N
55	529	ALB PROTEIN	IPI00708398			↓		69.5	5.82	extracellular space	transport	N
56	545	ALB PROTEIN	IPI00708398			↓		69.5	5.82	extracellular space	transport	N
57	561	U	U		↑			U	U	U	U	U
58	565	U	U		↑			U	U	U	U	U
59	587	U	U				↑	U	U	U	U	U
60	663	ALPHA-2-HS- GLYCOPROTEIN	IPI00707101			↓		38.4	5.25	extracellular space	acute-phase response; ossification; regulation of inflammatory response	cysteine-type endopeptidase inhibitor activity
61	669	ALPHA-2-HS- GLYCOPROTEIN	IPI00707101			↓		38.4	5.25	extracellular space	acute-phase response; ossification; regulation of inflammatory response	cysteine-type endopeptidase inhibitor activity
62	674	26 kDa PROTEIN	IPI00906471			↓		26.0	8.41	extracellular region	N	N
63	697	HSPA8 HEAT SHOCK COGNATE 71 kDa PROTEIN	IPI00708526			↓		71.2	5.37	cell surface; nucleolus; melanosome	response to stress; regulation of cell cycle	ATP binding; ATPase activity, coupled
64	700	ALPHA-1B- GLYCOPROTEIN	IPI00692686			↓		53.6	5.29	extracellular region	N	N
65	715	SEROTRANSFERRIN	IPI00690534			↓		77.8	6.75	extracellular region	cellular iron ion homeostasis	ferric iron binding
66	723	SEROTRANSFERRIN	IPI00690534			↓		77.8	6.75	extracellular region	cellular iron ion homeostasis	ferric iron binding
67	729	PIGR ISOFORM LONG OF POLYMERIC IMMUNOGLOBULIN RECEPTOR	IPI00696714		↑			82.4	7.07	extracellular region; plasma membrane	N	N
68	737	LACTOPEROXIDASE	IPI00716157			↓		80.6	8.83	extracellular space	defense response to bacteria; hydrogen peroxide catabolic process	heme binding; peroxidase activity
69	772	COMPLEMENT C3	IPI00713505		↑			187.3	6.41	extracellular space; extracellular region	inflammatory response; complement activation, alternative pathway	endopeptidase inhibitor activity
70	799	CATIONIC TRYPSIN	IPI00706427			↓		26.0	8.40	extracellular space	proteolysis; digestion	serine-type endopeptidase activity; metal ion binding

* Proteins not identified by MALDI-TOF/TOF; ^†^Upregulated proteins showing greater than 2-fold changes in the TC (LZ, LA and LF) and C groups compared to N group (*p*≤0.05); ^‡^No function annotation; ^§^Downregulated proteins showing greater than 2-fold changes in the TC and C groups compared to N group (*p*≤0.05).

**Figure 5:**
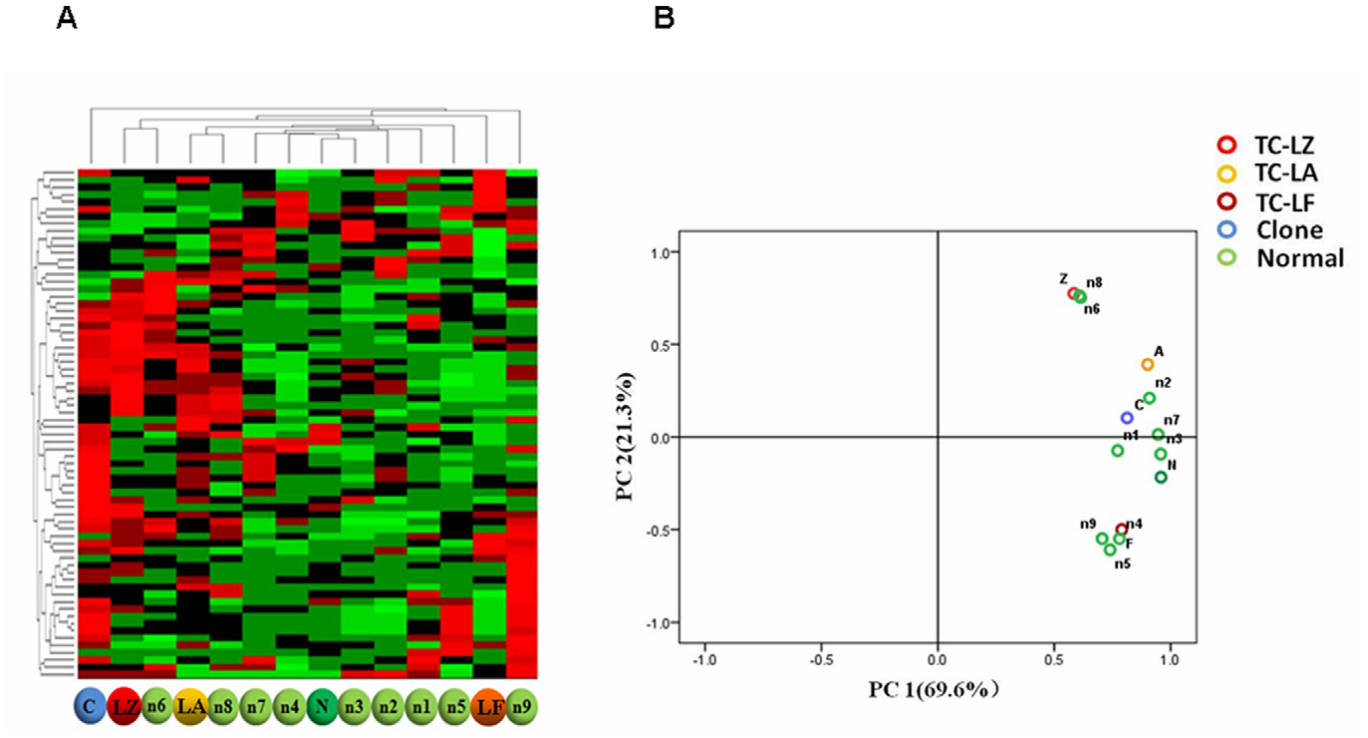
The intra- and inter-group relationships of the differentially expressed proteins of the TC, C and N groups. (A) HCA of differentially expressed proteins. (B) PCA of differentially expressed proteins.

An unsupervised hierarchical cluster analysis (HCA) was used to assess the effect of transgenes on, and the natural variability in, milk protein profiles from the TC-LZ, TC-LA, TC-LF, C and N groups in an unbiased manner. The heat map and hierarchical tree revealed that there was no difference between any TC group and the N group or between the direct clustering of the three TC groups and the C group (Figure 5A). The TC-LZ and TC-LA groups clustered with the normal samples n6 and n8, respectively. The TC-LF group clustered with samples n1-n8, and the TC-LZ and TC-LA groups both clustered with the normal sample n9. These results indicated that each TC group was closer to the normal samples than to the other TC groups or the C group.

A principal component analysis (PCA) was then used to analyze the significant differentially expressed spots from the 2D data. Specifically, there was no visible separation of any detected spots between the TC and N samples within the first two components (Figure 5B), with 69.6% of the variation allocated to the first component (PC1) and 21.3% of the variation allocated to the second component (PC2). These data showed a pattern largely similar to that generated by the HCA analysis, in which the three transgenes and their products showed very little impact on the milk protein profiles greater than that of natural variability. Thus, these data also suggest that the insertion of the three different exogenous human genes has no major impact on the general milk protein composition.

### Quantitative Comparison of Abundant Whey Proteins and Antibodies

An ELISA was used not only to obtain an accurate measurement of the concentration of the major highly abundant bovine whey proteins and immune-associated proteins (β-lactoglobulin, α-lactalbumin, serum albumin, transferrin, IgG, IgM and IgA) in the colostrum and mature milk for individual cows in the three TC groups and the N group but also to verify the quantitative proteomic results from the 2D PAGE statistical analyses.

**Figure 6:**
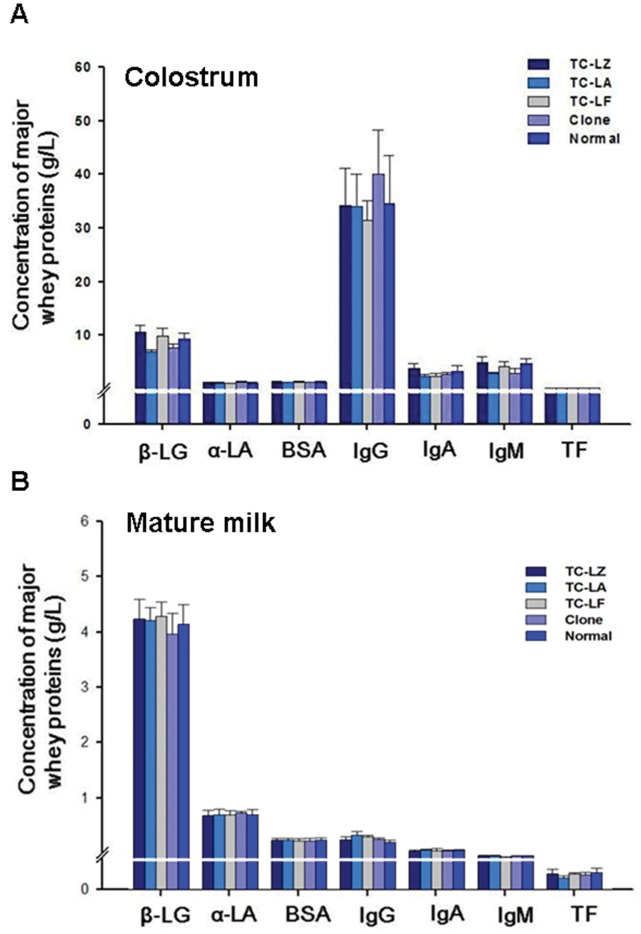
Comparisons of the major whey proteins and immune-associated proteins in the colostrum and mature milk from the TC, C, and N animals. Values are means ± SD and * indicate significant difference between the TC, C and N groups.

Overall, the concentrations of these major whey proteins, particularly the three most highly abundant whey proteins (β-lactoglobulin, α-lactalbumin and bovine serum albumin), were similar in the colostrum and mature milk and were not different between the TC, C and N groups (Figure 6). Although there were some minor fluctuations in the concentration of some proteins in the colostrum and mature milk of the TC groups, the range in the concentrations of the seven major whey proteins from the TC and C groups were not significantly different from those from the N group, which were largely within the bounds reported previously for common breeds of dairy cows in China. The results showed that the expression of additional human mammary gland-specific genes (α-lactalbumin, lactoferrin and lysozyme) in bovine mammary glands had minimal impact on the levels of the major whey proteins and immune-associated proteins, with all protein concentrations within the range of the natural variability observed in normal milk.

### Quantitative Comparison of Milk Whey Composition

**Figure 7:**
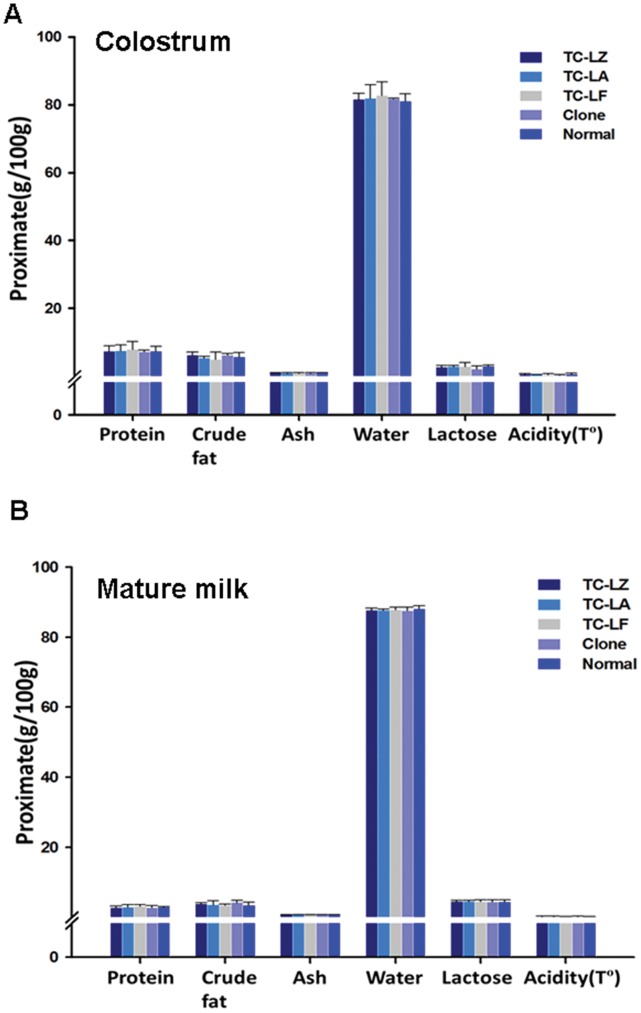
Comparisons of the proximates in the colostrum and mature milk from TC, C, and N animals. (A) The proximates of the colostrum from each group. (B) The proximates of the mature milk from each group. Values are means ± SD and * indicate significant difference between the TC, C and N groups.

**Figure 8:**
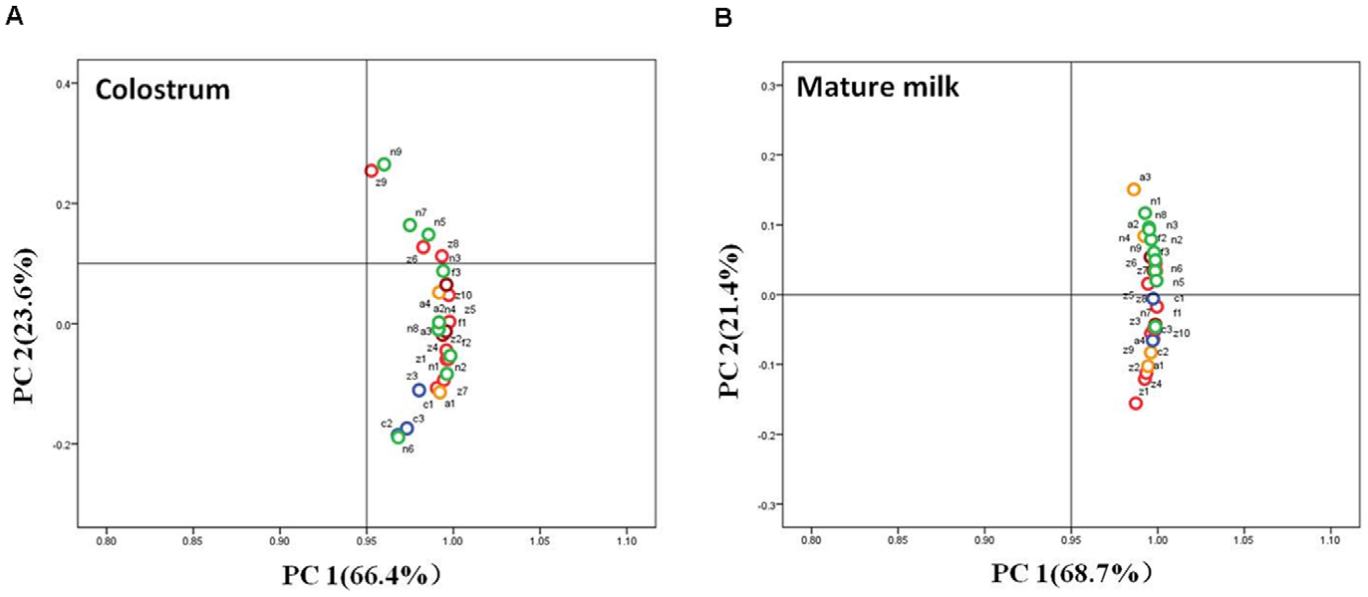
Graphical representation of the PCA results for the nutrient profiles of the colostrum and mature milk from TC, C, and N animals.

The major nutritional components of the colostrum and milk from the first three months of lactation from the TC, C and N groups were compared. First, the proximates analysis showed that the content of five principal milk nutrients of both the colostrum and milk from the first three months of lactation was very similar in all groups and was stable, which indicated that there are no obvious changes in the major components of milk among the groups (Figure 7). For a comparison of the micro composition, more than 50 nutritional components were analyzed in the colostrum and mature milk of all the groups, and more than 90% of these components showed no significant difference among the groups (Figure S1–S5). However, there was a minor variation in 7 components compared with the N group (*p*≤0.05) as follows: C16∶1 in the mature milk of TC-LF; C20∶4n6 in the mature milk of C; Mg in the colostrum and mature milk of C and TC-LA, respectively; Se and K in the mature milk of TC-LZ; and vitamin C in the mature milk of TC-LZ and TC-LF. PCA was used for multivariate analyses of the intra- and inter-group relationships among the TC, C and N groups. In both the colostrum and mature milk, clusters were observed in the first two principal components, which accounted for 66.4% and 23.6%, 68.7% and 21.4%, respectively. This finding indicates that the nutrient levels in the TC and C groups are within the range of natural variability (Figure 8).

## Discussion

Although the use of transgenic cloning technology to increase the expression of recombinant proteins in the mammary glands has been successful, changes in intermolecular interactions caused by the process of cloning transgenic animals are not yet fully understood. Traditional analytical methods used to detect specific components of transgenic food (such as nutritional substances, allergens and other nutrients) are incapable of effectively assessing potentially unpredictable changes. Some findings suggest that somatic cloning technology does not induce changes in the main nutrient content of cloned beef and milk [8], [22]–[25]. However, thus far, there have been no reports using proteomics to comprehensively analyze milk from transgenic animals. Here, we analyzed the protein composition and nutrient components of the colostrum and mature milk from 17 transgenic cloned cows expressing one of three mammary gland-specific genes, 3 cloned cows and 9 normal cows using proteomics and metabolomics to analyze the maximum number of unpredictable changes in the milk from TC animals. The transgenes in the three transgenic cloned cattle breeds were stably integrated into random positions in the genome and specifically expressed in mammary gland tissue. The recombinant hα-LA gene and hLF gene were more highly expressed in the corresponding TC group than in the N and C groups, with an average expression level of approximately 1.5 mg/mL and 3.5 mg/mL, respectively, and the hLZ gene was expressed in transgenic cows at approximately 0.01 mg/mL. MALDI-TOF mass spectrometry showed that these three recombinant human proteins had the same molecular weight and N-terminal amino acid residue protein sequence as the endogenous homologous genes.

The merged data from previous investigations showed that an exhaustive list of 284 non-redundant annotated protein entries derived from whey proteins [26]. In this study, a total of 301 ordinary milk whey proteins were characterized in all groups. To the best of our knowledge, this study is the most comprehensive characterization of mature milk proteins in bovine whey to date. None of the 37 newly identified proteins in the TC groups was common to all three groups, which indicated that during the transgenic cloning process, potential factors, such as epigenetic errors, did not cause the specific expression of proteins in the transgenic cloned milk. In addition, we found that the 37 specifically expressed proteins identified by LC-MS were not detected in the full milk protein profile of the subsequent 2DE combined with MALDI-TOF/TOF. The most obvious difference between 2DE and the LC-MS technique is that it is more difficult to separate hydrophobic, extremely acidic or basic proteins by LC-MS [27]. However, in this experimental analysis, the milk proteins in the samples were water-soluble proteins, and their isoelectric points were distributed between pH 4 and pH 7. Consequently, when the milk proteome was isolated and identified, the only difference between the two techniques was the successful separation of low-abundance proteins [28]. In addition, the smallest detected protein spots on “blue-silver” staining gels have been demonstrated to contain 1 ng of protein; thus, this staining method approaches the sensitivity of silver staining [29]. Moreover, silver staining, which is the most sensitive protein detection technique, was also used in an attempt to identify the specifically expressed proteins, but these proteins were unfortunately still beyond the detection range [30]. Therefore, we inferred that the 37 specifically expressed proteins in the TC groups were of trace abundance.

There are biases in the identification of low-abundance proteins using LC-MS, and the success rate of identifying proteins of low abundance is low because the peptides that are produced enzymatically from proteins of high and low abundances cannot be completely separated by the 2D separation step in LC and the peptides derived from high level proteins may disturb the mass spectrum signal of the peptides from the low-abundance proteins [31]. In this study, the MS identification of five whey samples was repeated 3 times under the same conditions and using the same instrument to improve the sensitivity, comprehensive coverage and reproducibility of detection. In addition, the replicates were also made to counterbalance the extreme complexity and large dynamic range of the protein components of milk. However, there were still 7 proteins that were specifically expressed in the TC groups and were also identified in the previous report of the profile of milk from conventionally bred cattle, indicating the inevitable inability to identify low-abundance milk proteins. This observation demonstrates that not all these proteins can be considered to be truly specifically expressed in the TC groups owing to the limitations on identifying proteins of trace abundance, which are beyond the detection range.

For the differentially expressed proteins, the successfully identified 46 protein spots were classified into 22 types of proteins as one protein can be present in different spots because of glycosylation or degradation. The concentration of SERPINB1 was significantly changed in all four groups. It has been categorized as a peptidase inhibitor and is thought to reduce protein digestibility and allow proteins to reach the intestinal tract. Most of the differentially expressed proteins are involved in biological processes with the partial function of defense response, providing protection against infection. This function includes 6 out of 13 identified proteins that have significantly higher expression levels, mostly in the C group, such CATHL1, complement C3, α-1-acid glycoprotein (AGP), zinc-α-2-glycoprotein (AZGP1), BOLA-NC1 and ORM1. Our data found no difference in the expression of immunological proteins, such as IgG, IgA and IgM, in milk between the different groups, which suggests that these immunological factors play a role in the defense mechanism while not directly influencing the milk protein components. In addition, lactoperoxidase (LPO) and alpha-2-HS-glycoprotein (AHSG), which are involved in the defense response, were downregulated in TC-LF milk, which might indicate that human lactoferrin in large amount plays a crucial role in immune defense. PCA and HCA analysis showed that the variation in the protein profile in the TC groups was within the limits of natural variability. These data illustrate that the expression of heterologous proteins did not significantly impact the milk whey protein profile.

Recombinant proteins expressed in these transgenic cattle are human milk proteins that will be consumed by humans. Levels of the major whey proteins were stable and not significantly different in the colostrum or ordinary milk among different groups, which were similar to those from the quantitative analyses using 2DE. The high expression levels of hLF in milk may have caused the decreased expression of low-abundance endogenous proteins but may not have affected the levels of highly abundant proteins. No such relationship was apparent in the TC-LA and TC-LZ groups. Thus, we deduced that there were no predictable relationships between the expression of human milk proteins and the expression of endogenous proteins in the three TC groups and therefore that there was no effect on the expression of the endogenous genes. Milk is a complex biological fluid, and the milk proteome is a dynamic entity that is influenced by environmental, genetic and epigenetic effects. In addition, the period of lactation makes a small difference, so the milk components will change with the lactation period. In this study, we analyzed more than 50 parameters representing the major nutritional components of milk. The quantity of every parameter within each group and among groups was variable; however, most of these parameters were relatively stable, the values did not significantly change from those of the normal group. In addition, we found that all the groups showed similar and normal lactation curves. A small number of the parameters had significant changes among the groups, but the difference among the groups was not greater than that within the groups, indicating that they may be due to individual differences, not genetic modification.

In summary, this study is the first attempt to use proteomic approaches to provide more comprehensive information on the milk proteome. The expression of exogenous proteins did not significantly change the milk whey protein profile, and the mean values for the majority of the measured parameters were all within the normal range. The differences among the groups were not greater than those within the groups, i.e., the differences were within the scope of intragroup variability, indicating that the differences were due to individual differences between cattle, not genetic modification. It is important to note that this study was conducted with a small number of transgenic cloned cattle. The information obtained from these results, however, will improve the understanding of the bovine milk proteome and provide data for the assessment of the food safety of transgenic cloned animals, which is expected to increase the acceptance of consumers.

## Supporting Information

Figure S1 **Comparisons of the amino acid content of the colostrum and mature milk from TC, C, and N animals.** Values are means ± SD and * indicate significant difference between the TC, C and N groups.
(TIF)

Figure S2 **Comparisons of the fatty acid content of the colostrum from TC, C, and N animals.** Values are means ± SD and * indicate significant difference between the TC, C and N groups.
(TIF)

Figure S3 **Comparisons of the fatty acid content of the mature milk from TC, C, and N animals.** Values are means ± SD and * indicate significant difference between the TC, C and N groups.
(TIF)

Figure S4 **Comparisons of the mineral content of the colostrum and mature milk from TC, C, and N animals.** Values are means ± SD and * indicate significant difference between the TC, C and N groups.
(TIF)

Figure S5 **Comparisons of the vitamin content of the colostrum and mature milk from TC, C, and N animals.** Values are means ± SD and * indicate significant difference between the TC, C and N groups.
(TIF)
