# Supplementary figures and images for: Comprehensive Assessment of Milk Composition in Transgenic Cloned Cattle
Source: PLoS One. 2012 Nov 21;7(11):e49697. doi: 10.1371/journal.pone.0049697 (PMC3504162; doi:10.1371/journal.pone.0049697)

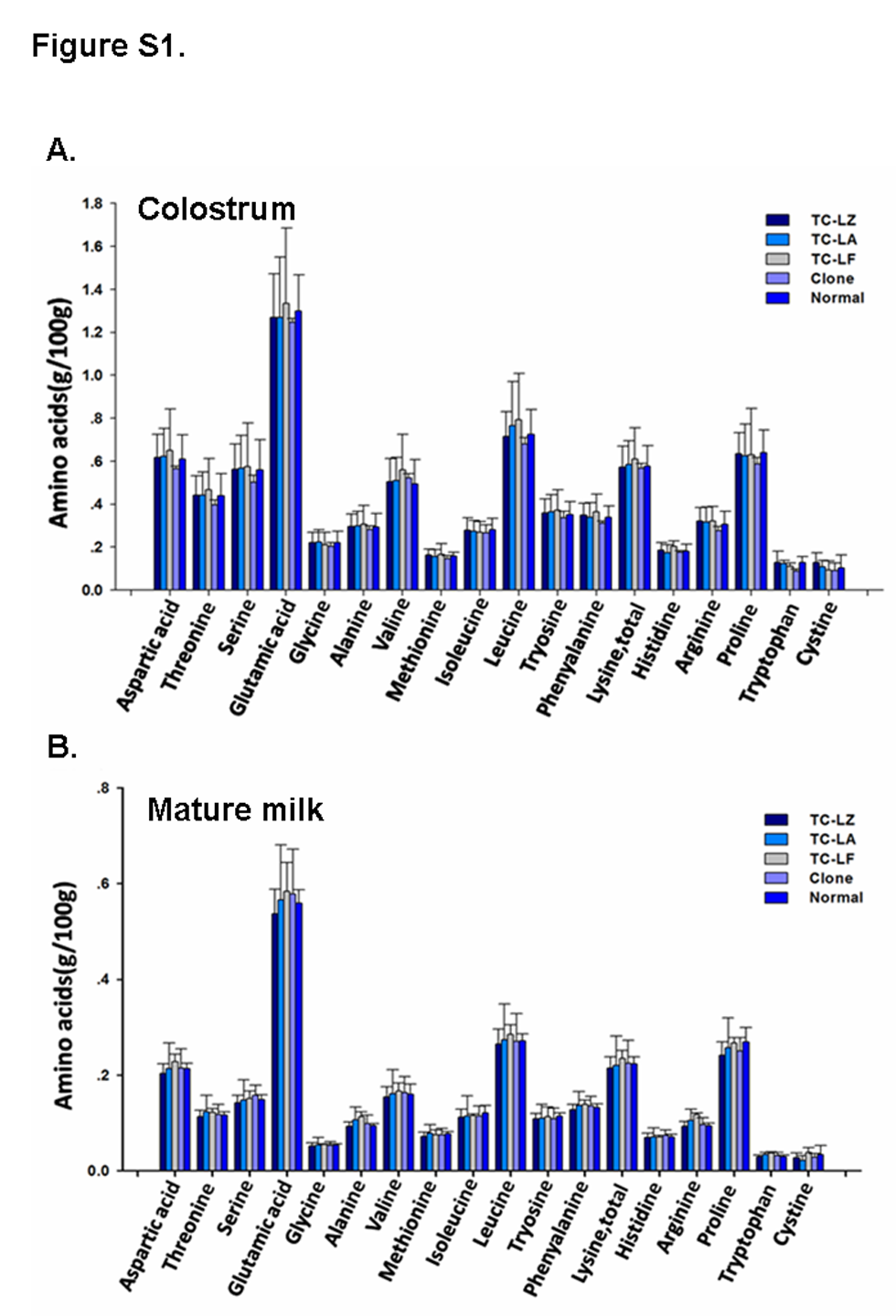

Supplement: Figure S1 — Comparisons of the amino acid content of the colostrum and mature milk from TC, C, and N animals. Values are means ± SD and * indicate significant difference between the TC, C and N groups. (TIF) [file pone.0049697.s001.tif]

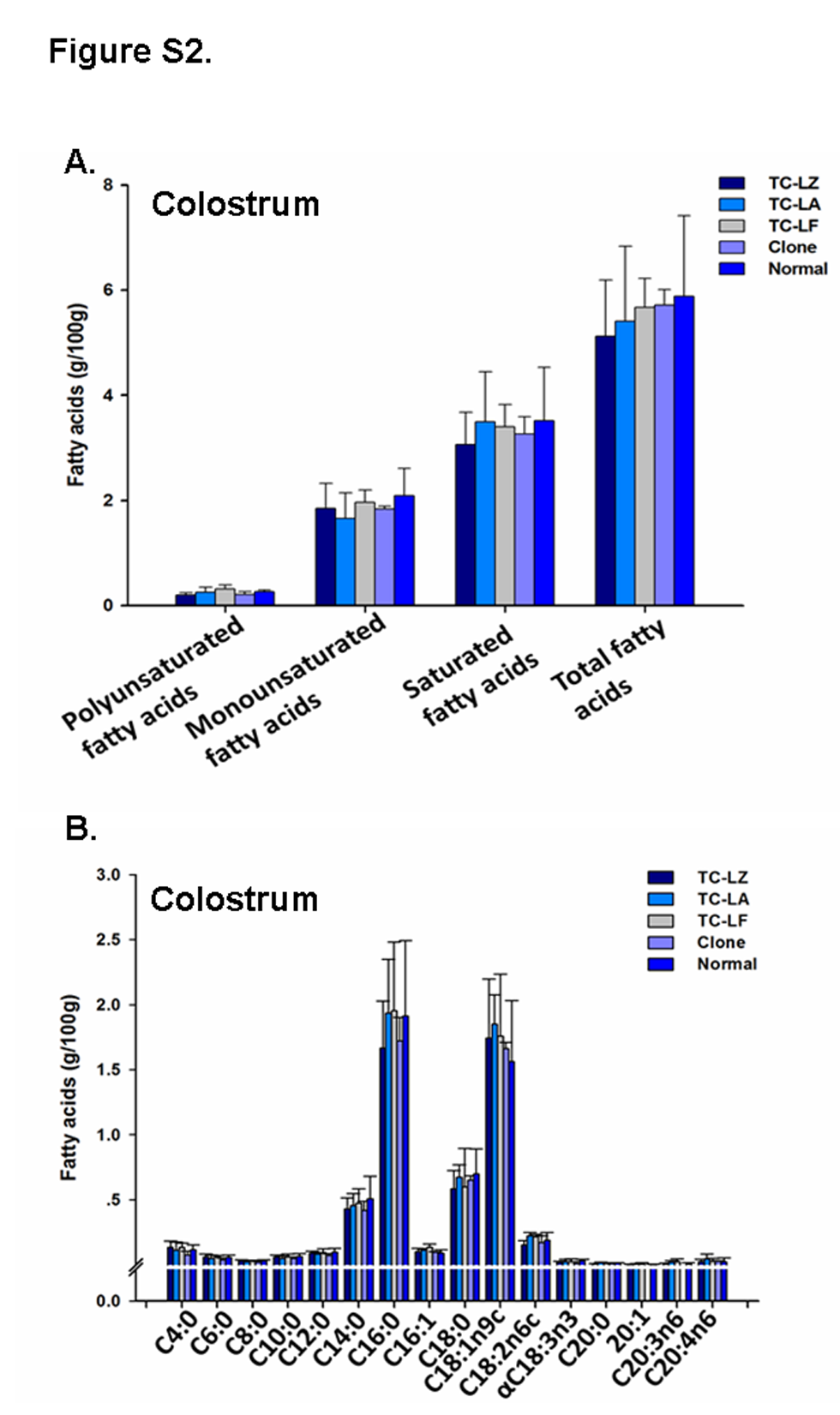

Supplement: Figure S2 — Comparisons of the fatty acid content of the colostrum from TC, C, and N animals. Values are means ± SD and * indicate significant difference between the TC, C and N groups. (TIF) [file pone.0049697.s002.tif]

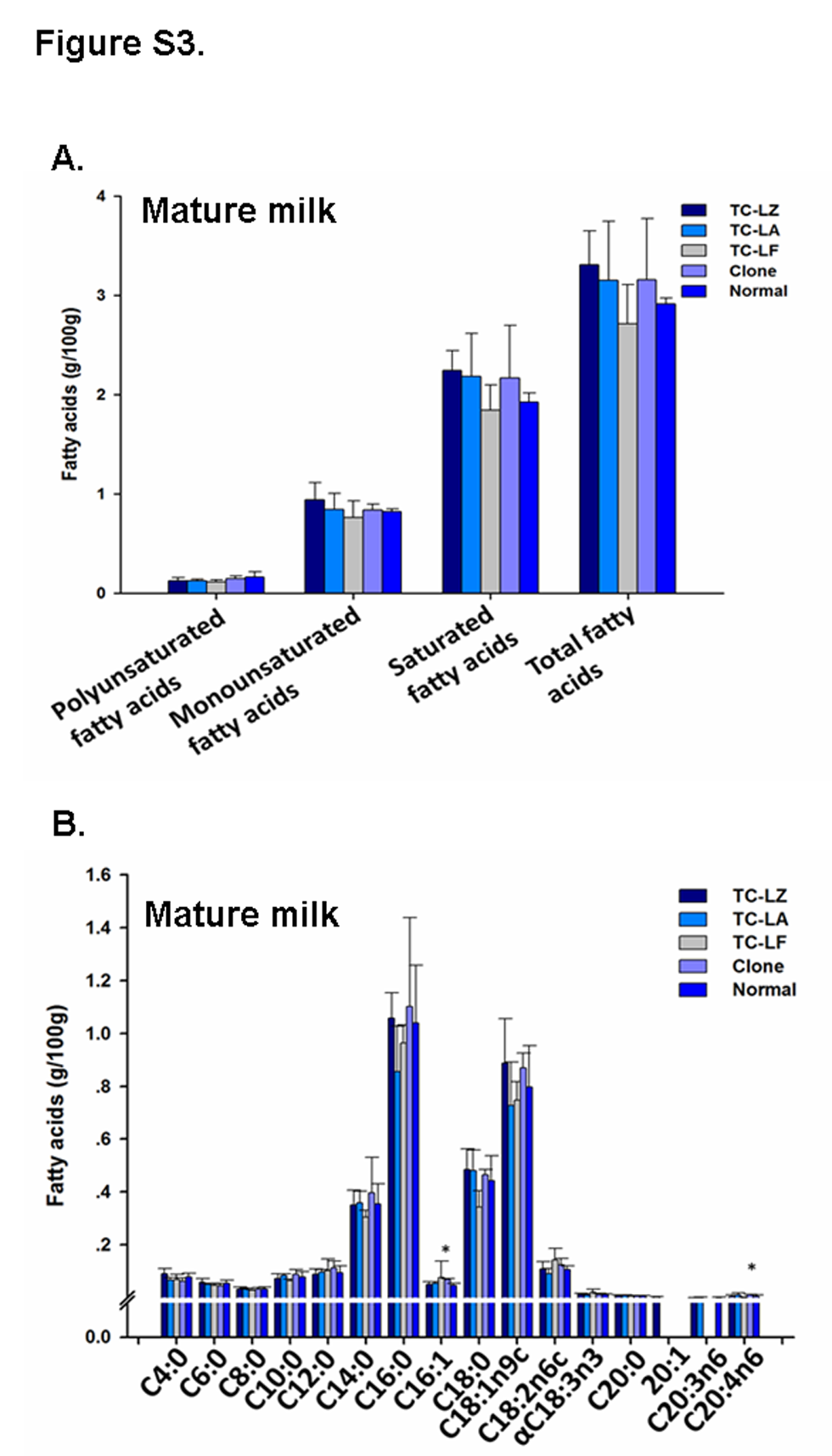

Supplement: Figure S3 — Comparisons of the fatty acid content of the mature milk from TC, C, and N animals. Values are means ± SD and * indicate significant difference between the TC, C and N groups. (TIF) [file pone.0049697.s003.tif]

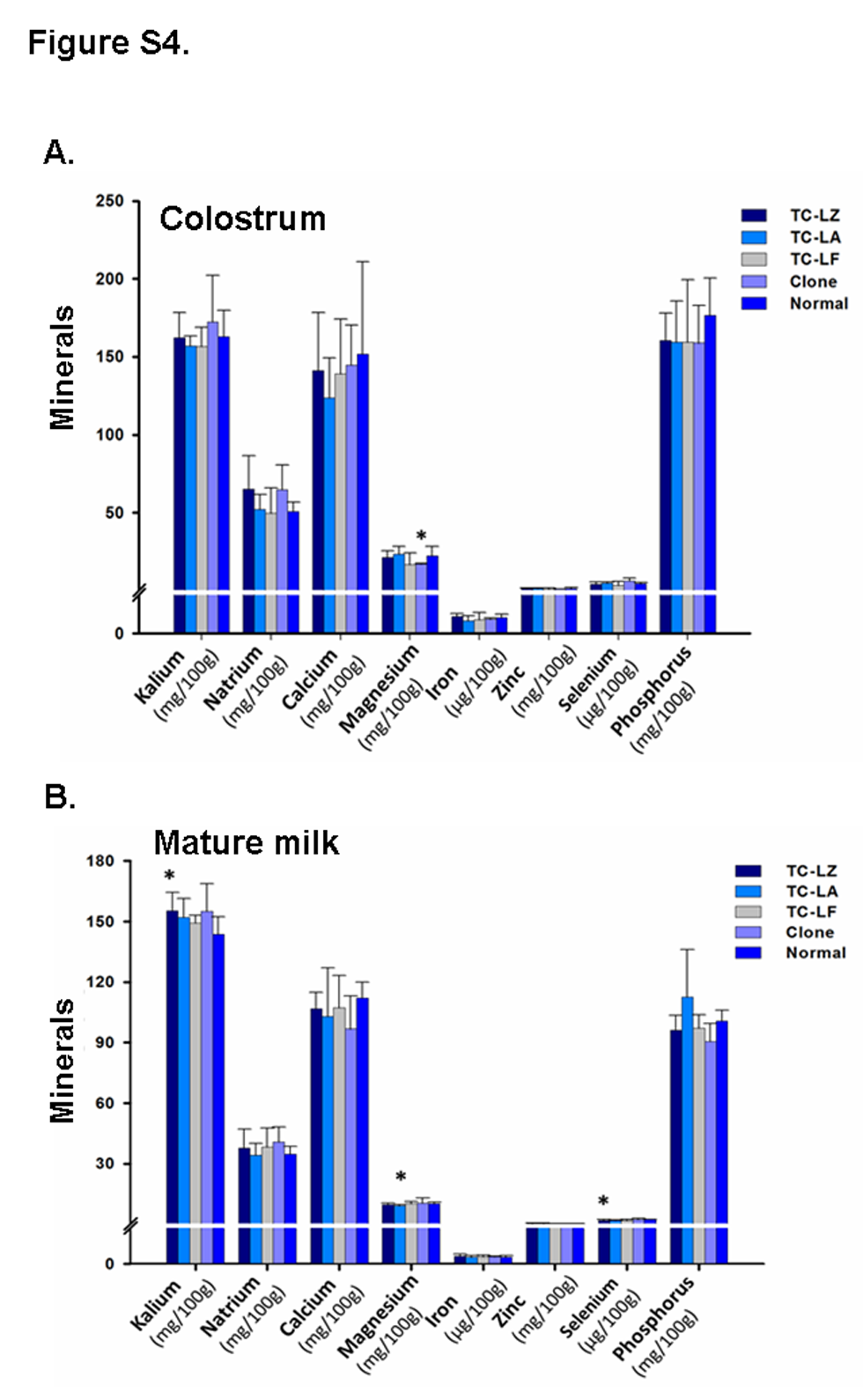

Supplement: Figure S4 — Comparisons of the mineral content of the colostrum and mature milk from TC, C, and N animals. Values are means ± SD and * indicate significant difference between the TC, C and N groups. (TIF) [file pone.0049697.s004.tif]

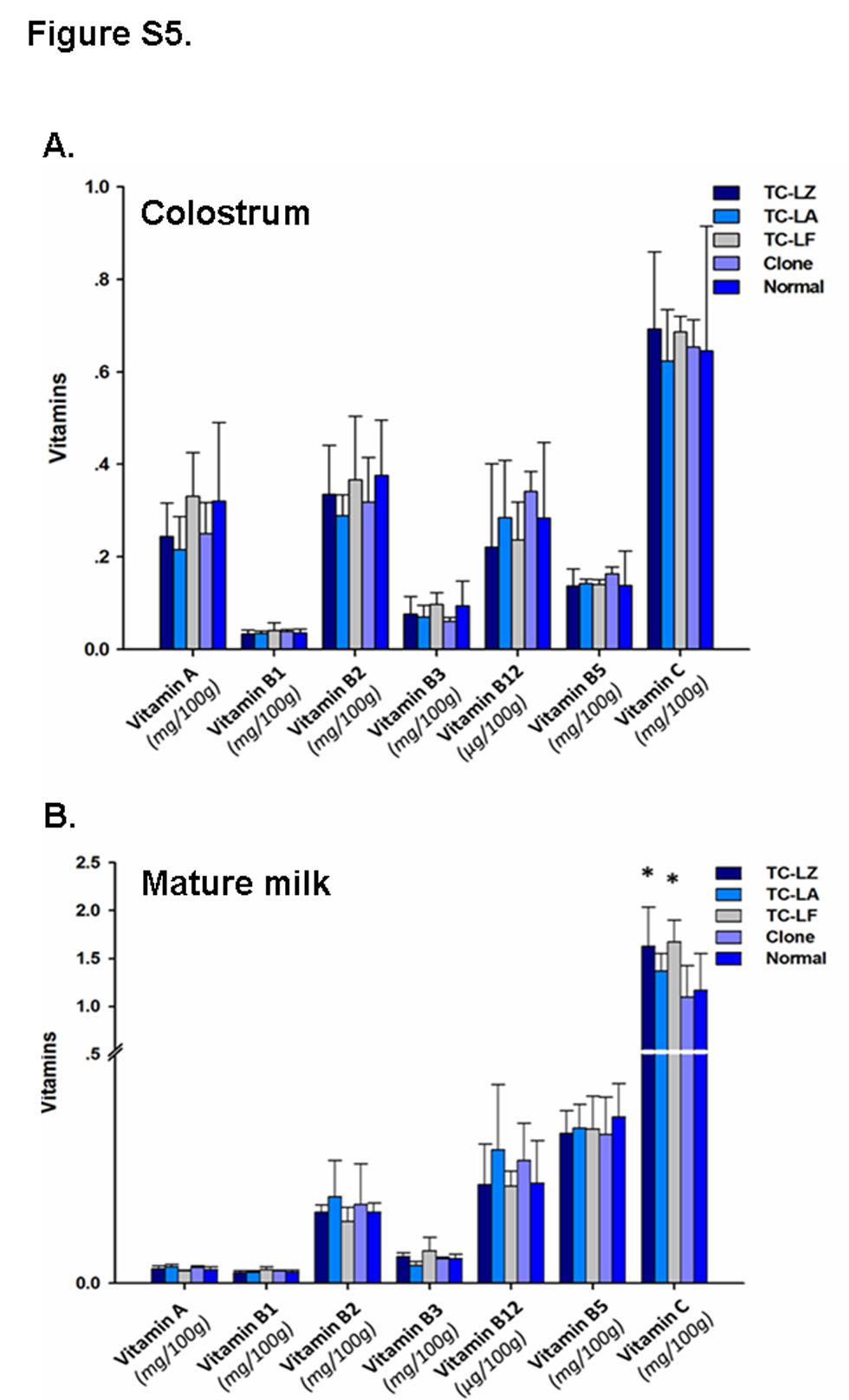

Supplement: Figure S5 — Comparisons of the vitamin content of the colostrum and mature milk from TC, C, and N animals. Values are means ± SD and * indicate significant difference between the TC, C and N groups. (TIF) [file pone.0049697.s005.tif]
